# Development and utilization of new O_2_-independent bioreporters

**DOI:** 10.1128/spectrum.04091-23

**Published:** 2024-03-05

**Authors:** Eva Agranier, Pauline Crétin, Aurélie Joublin-Delavat, Léa Veillard, Katia Touahri, François Delavat

**Affiliations:** 1 Nantes Université, CNRS, US2B, UMR6286, Nantes, France; 2 Laboratoire Chimie et Biochimie de Molécules Bioactives, Université de Strasbourg/CNRS, UMR7177, Strasbourg, France; The Pennsylvania State University, University Park, Pennsylvania, USA

**Keywords:** bioreporter, flavin-binding fluorescent protein, *Vibrio*, *Rhizobiaceae*, anaerobic condition, anoxia

## Abstract

Fluorescent proteins have revolutionized science since their discovery in 1962. They have enabled imaging experiments to decipher the function of proteins, cells, and organisms, as well as gene regulation. Green fluorescent protein and all its derivatives are now standard tools in cell biology, immunology, molecular biology, and microbiology laboratories around the world. A common feature of these proteins is their dioxygen (O_2_)-dependent maturation allowing fluorescence, which precludes their use in anoxic contexts. In this work, we report the development and *in cellulo* characterization of genetic circuits encoding the O_2_-independent KOFP-7 protein, a flavin-binding fluorescent protein. We have optimized the genetic circuit for high bacterial fluorescence at population and single-cell level, implemented this circuit in various plasmids differing in host range, and quantified their fluorescence under both aerobic and anaerobic conditions. Finally, we showed that KOFP-7-based constructions can be used to produce fluorescing cells of *Vibrio diazotrophicus*, a facultative anaerobe, demonstrating the usefulness of the genetic circuits for various anaerobic bacteria. These genetic circuits can thus be modified at will, both to solve basic and applied research questions, opening a highway to shed light on the obscure anaerobic world.

**IMPORTANCE:**

Fluorescent proteins are used for decades, and have allowed major discoveries in biology in a wide variety of fields, and are used in environmental as well as clinical contexts. Green fluorescent protein (GFP) and all its derivatives share a common feature: they rely on the presence of dioxygen (O_2_) for protein maturation and fluorescence. This dependency precludes their use in anoxic environments. Here, we constructed a series of genetic circuits allowing production of KOFP-7, an O_2_-independant flavin-binding fluorescent protein. We demonstrated that *Escherichia coli* cells producing KOFP-7 are fluorescent, both at the population and single-cell levels. Importantly, we showed that, unlike cells producing GFP, cells producing KOFP-7 are fluorescent in anoxia. Finally, we demonstrated that *Vibrio diazotrophicus* NS1, a facultative anaerobe, is fluorescent in the absence of O_2_ when KOFP-7 is produced. Altogether, the development of new genetic circuits allowing O_2_-independent fluorescence will open new perspective to study anaerobic processes.

## INTRODUCTION

Fluorescent proteins have revolutionized our understanding of many biological systems, allowing direct visualization of events on a wide range of spatiotemporal scales. GFP (green fluorescent protein), discovered fortuitously in 1962 (1) and its numerous derivatives [RFP, CFP, YFP, EGFP (Enhanced GFP), mTurquoise, mCherry, etc. (2)] have been particularly used as reporter proteins to address many fundamental and applied questions related to cell visualization, protein-protein interactions, protein localization, biosensor construction, biofilm formation, and gene expression dynamics. One of its major advantages relies in the fact that chromophore maturation does not require any cofactor or enzyme, implying that producing the protein *in cellulo* is sufficient to fluorescently tag cells or cell components. However, the main drawback of these proteins is that they require molecular dioxygen (O_2_), which dehydrogenates a critical tyrosine residue to form the chromophore, allowing fluorescence (3). This dependence on O_2_ is a major hindrance to the characterization of systems under low O_2_ tension conditions.

On the other hand, many key microbial metabolic processes occur in hypoxia or anoxia. These can be found in environmental contexts, such as soil (4), deep and costal sea waters (5), or eutrophic lake waters (6), and infectious contexts such as gastrointestinal tracts (7) or tumors (8). In these O_2_-deprived environments, the use of GFP or derivatives is therefore inefficient. In order to circumvent this pitfall, organic dyes have been experimentally tested, but those reveal poorly permeable and/or are cytotoxic, and often accompanied by a high fluorescence background [for a review, see reference (9)]. Fluorescent proteins have also been used in anoxia. For example, strict anaerobic bacteria such as *Clostridioides difficile* producing the fluorescent protein mCherry can fluoresce when samples are placed in the open air for 1 hour (10). However, this dependence on oxygen can lead to cell lysis in strict anaerobes (10), and does not allow its use in real time to monitor the dynamics of biological processes. Other O_2_-independent fluorescent proteins exist, such as UnaG (11), which uses bilirubin as a cofactor to fluoresce (12). However, they require the addition of this cofactor in the culture medium, making these reporter genes of little interest in complex contexts such as the *in vivo* study of bacteria interacting with its host (symbioses, pathogens). Similarly, *lux* bioluminescence genes, although they can be applied to anaerobes (13), do not allow quantification of the expression of genes of interest at the single-cell level.

Recently, another class of reporter genes has emerged, and these genes encode flavin-binding fluorescent proteins (FbFPs) (14, 15). FbFPs are derived from a large family of proteins called LOV (light, oxygen, and voltage) proteins. LOV proteins possess a flavin mononucleotide (FMN)-binding pocket containing a cysteine residue in which the FMN (which possesses intrinsic fluorescence properties) will bind to form a covalent adduct. Substituting the cysteine residue to alanine triggers modifications, ultimately leading to stable protein fluorescence emission (14, 16). FbFPs present many advantages: they are small (100–140 amino acids, compared to ~240 amino acids for GFP), have a fast maturation time,and fluoresce at similar wavelengths as GFP (16). Moreover, they have the great advantage of not requiring O_2_ to mature and fluoresce. This O_2_-independent maturation opens the way to the development of new reporter genes for the study of biological systems under anoxic or variable O_2_ tension conditions. Several developed FbFP derivatives have allowed the study of gene expression of subcellular localization of proteins in different prokaryotic and eukaryotic models (14, 15). However, these bioreporter proteins typically exhibit low fluorescence compared to GFP and its derivatives [about 30-fold (17)], and this represents a bottleneck for their application. An FbFP called phiLOV was reported to be fluorescent in the obligate anaerobe *C. difficile* (18), but cell fluorescence remains low (19).

Recently, a random mutagenesis approach was conducted on a gene encoding the SB2 protein (14), an FbFP from *Pseudomonas putida*. After several rounds of enrichment in fluorescent clones, the KOFP-7 protein was obtained (17). The *in vitro* purified KOFP-7 protein shows a fluorescence intensity as high as that of GFP, as well as a good stability in acidic media or in the presence of reducing agents. These characteristics make KOFP-7 a good candidate for the development of new reporter genes for application to the study of microbial biological systems under anoxic conditions. However, whether KOFP-7 can be used *in vivo* remains to be demonstrated.

In this study, we present the generation of a series of replicative plasmids, differing in their genetic circuits and bacterial hosts, improved for KOFP-7 production and fluorescence under anaerobic conditions. Moreover, we demonstrated that KOFP-7-based fluorescence can be obtained both in *Escherichia coli* and in *Vibrio diazotrophicus,* demonstrating the versatility of these construction. These genetic circuits will contribute in shedding a new light in previously dimmed anaerobic microbes, for future basic and applied discoveries.

## RESULTS

### Constructions with KOFP-7 allow *in cellulo* fluorescence under aerobic conditions

**Fig 1:**
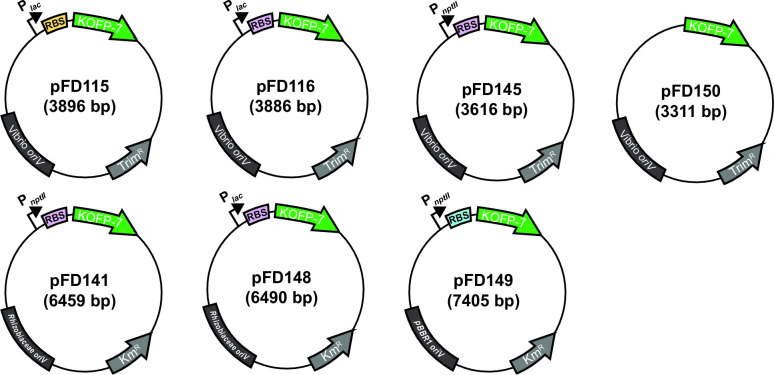
Schematic overview of the constructed plasmids. Identical colors indicate identical sequences.*oriV*, origin of replication.

In order to test the possibility of using KOFP-7 as a new reporter protein, we sought to create various plasmids, differing in their ribosome binding site (RBS) region, promoter sequences, as well as backbone plasmids, all being designed to constitutively express *kofp-7*. We created seven plasmids (Fig. 1), all of them being able to replicate in *E. coli*, among which four can replicate in *Vibrionaceae* and *Pseudoalteromonaceae* families (20, 21) (pFD115, pFD116, pFD145, and pFD150, the latter being promoterless and serving as a negative control), two being able to replicate in *Rhizobiaceae* (pFD141 and pFD148), and one containing the broad host-range origin of replication pBBR1 (22) (pFD149).

**Fig 2:**
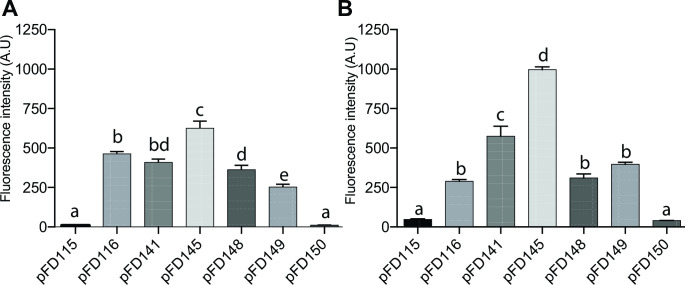
Population-based fluorescence from *kofp-7*-containing strains, measured by fluorimetry after growth under aerobic condition. (**A**) Fluorescence of *E. coli* carrying the different plasmids measured at time 0. (**B**) Fluorescence of *E*. coli carrying the different plasmids measured after 5 hours of growth. Presented here are the mean + SEM.

Our initial screening was performed by quantifying the fluorescence of each of these populations in microplates in the presence of oxygen, all being diluted to the same Optical Density (OD) from overnight cultures. We showed here that while pFD115 fails to produce a fluorescence signal above background, all other constructions allowed fluorescence already at time 0, when cells were in stationary phase (Fig. 2A). Similar conclusions were obtained when the plates were incubated for 5 hours at 37°C before measurement (Fig. 2B), despite all still having a similar final OD. It is worth noting that pFD115 and pFD116 are very similar, differing only in their RBS region, but the measured fluorescence intensity greatly differs. The highest fluorescence was obtained with the *E. coli* strain carrying pFD145, a plasmid which carries the P*_nptII_* promoter instead of the P*_lac_* promoter found in pFD116, the RBS region originally found in pFD086 and the *kofp-7* gene. Interestingly, pFD116 and pFD145 vary only in their promoter sequence, but the measured fluorescence intensity was 3.3-fold higher with the strain containing pFD145 at time 5 hours. This shows that the P*_nptII_* promoter is stronger than P*_lac_* in this genetic context.

pFD141 and pFD148, both having the same structure and differing only in the inserted promoter (P*_nptII_* and P*_lac_*, respectively), show intermediate fluorescence intensity, with pFD141 allowing a slightly higher KOFP-7 production (despite being non-significant at time 0). This confirms that P*_nptII_* is a stronger promoter, also in this genetic context. Finally, the broad-host range vector pFD149, endowed with the P*_nptII_* promoter shows intermediate results, with the fluorescence being higher than pFD115 and lower than the others at time 0, and similar to pFD116 and pFD148 at time 5 hours.

In conclusion, we demonstrate here that the strains containing *kofp-7* as a reporter gene are fluorescent when measured at the population level in microplate. The next step was to quantify the fluorescence level of single cells, using epifluorescence microscopy equipped with a regular Fluorescein IsothioCyanate (FITC)-based filter cube.

### KOFP-7-based bioreporters are fluorescent in single bacterial cells

**Fig 3:**
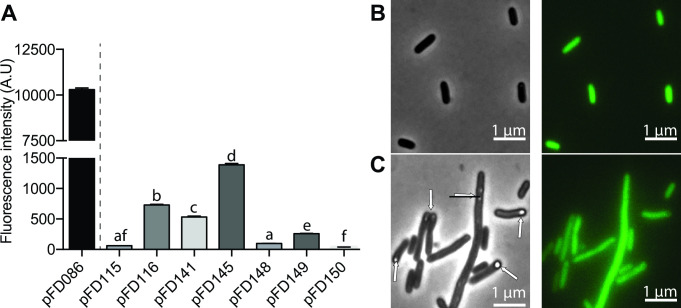
Single cell-based fluorescence from *kofp-7*-containing strains after growth under aerobic condition. (**A**) Quantification by epifluorescence microscopy under aerobic condition. Statistical analysis was performed between all *kofp-7*-containing strains. Presented here are the mean + SEM. Letters above each histogram represent their statistical grouping determined by a one-way analysis of variance with Tukey test. (**B**) *E. coli* cells carrying pFD145 and having a classical rod shape. (**C**) *E. coli* cells carrying pFD141 and showing aberrant elongated cells, with structures resembling inclusion bodies (see white arrows). Images were scaled to the same brightness.

Bioreporters are particularly useful to monitor metabolic processes or to follow the fate of single bacterial cells under various conditions, and epifluorescence microscopy is the corresponding gold standard. Demonstrating that the newly designed bioreporters can be used at the single-cell level was therefore necessary. Cells were grown overnight in Lysogenic Broth (LB) supplemented with their corresponding antibiotics under aerobic conditions, and fluorescence of single bacterial cells was quantified using in-house Matlab scripts (23). Under this condition, single-cell fluorescence measurements are in total accordance with the fluorescence measured from whole populations in microplate (Fig. 3A). Specifically, *E. coli* carrying pFD145 performed best, showing the highest per-cell fluorescence intensity, followed by the strains carrying pFD116 and pFD141. Again, *E. coli* cells carrying pFD115 were not fluorescent. This shows that the increase in fluorescence observed in microplate observed for most constructions (Fig. 2) is mostly due to the genetic circuit allowing KOFP-7 production, which differs between plasmids. Under this aerobic conditions, *E. coli* carrying pFD086, differing from pFD116 by the production of EGFP instead of KOFP-7, shows a much higher per-cell fluorescence, as expected from an optimized fluorescent molecule (Fig. 3A). Of note, *E. coli* cells carrying pFD115, pFD116, pFD145, and pFD149 are rod-shaped, as expected from *E. coli* cells (Fig. 3B). However, some cells of strains carrying pFD141 and pFD148 are slightly elongated, possibly reflecting a bacterial stress (Fig. 3C). These cells also show intracellular structure resembling inclusion bodies (Fig. 3C).

**Fig 4:**
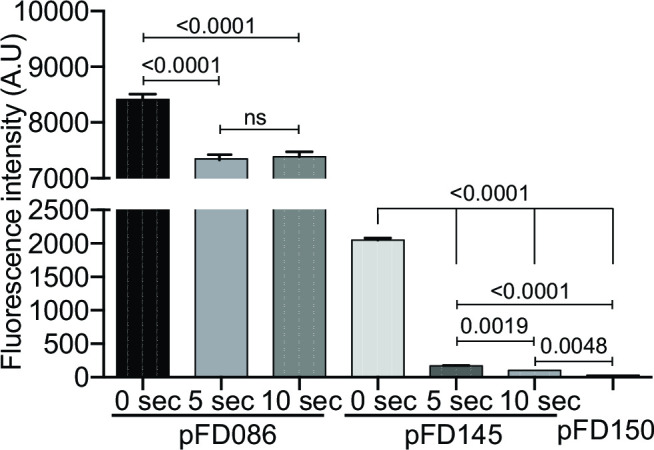
Rapid photobleaching of KOFP-7. Strains producing either the EGFP (pFD086) or KOFP-7 (pFD145) were exposed for 5 to 10 seconds of continuous light, under a microscope equipped with a 482 +/− 35 nm filter cube. Fluorescence intensity of single cells was measured at time 0 and after the exposure time. Results from pFD145 were compared to the results of the negative control (strain carrying the promoterless plasmid pFD150). ns, not significant.

Finally, we wanted to know the *in cellulo* stability of KOFP-7 fluorescence and its propensity of photobleaching. Therefore, *E. coli* cells carrying pFD145 were deposited on an agarose patch, and cells were exposed to continuous excitation at 482 +/− 35 nm. Strikingly, cells experienced rapid photobleaching, with cell fluorescence decreasing sharply within a few seconds, the decrease being visible under the microscope oculars. Photobleaching was measured by quantifying cell fluorescence at time 0 and the fluorescence of the same cells after 5 or 10 seconds of continuous exposure. Cell fluorescence decreased rapidly, reaching a 21-fold decrease after 10 seconds, almost returning to background fluorescence observed from cells carrying the promoterless plasmid pFD150 (Fig. 4). Cells carrying pFD086 (and thus producing GFP) exposed to the same treatment were in contrast much less photobleached, with a fluorescence decreasing only 1.1-fold (Fig. 4).

### Constructions with KOFP-7 allow *in cellulo* fluorescence under anaerobic conditions

**Fig 5:**
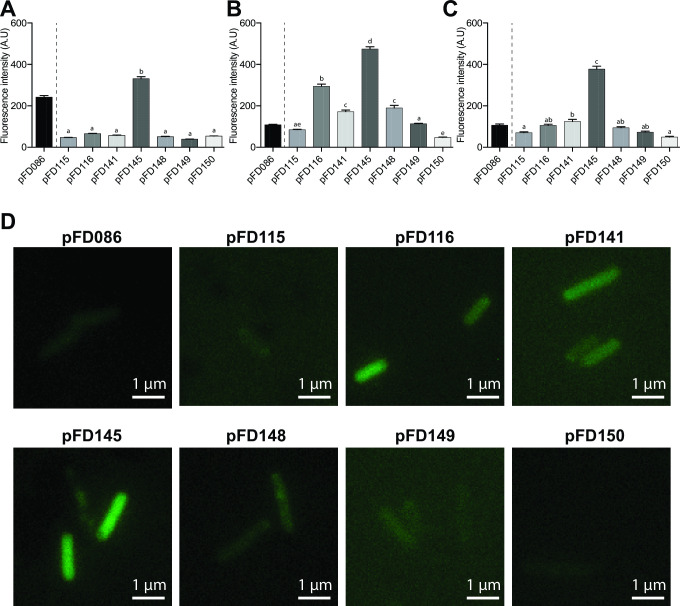
Single cell-based fluorescence from *kofp-7*-containing strains after growth under anaerobic conditions measured by epifluorescence microscopy. Cells were grown (**A**) in anaerobic LB conditions, (**B**) in anaerobic LB containing NO_3_^-^ as an alternative electron acceptor, and (**C**) in anaerobic LB containing glucose as a fermentative carbon source. Statistical analyses were performed between all *kofp-7*-containing strains. Presented here are the mean + SEM. Letters above each histogram represent their statistical grouping determined by a one-way analysis of variance with Tukey test. (**D**) Illustration of the fluorescence of single cells. Images were scaled to the same brightness.

In order to quantify the fluorescence of the different constructs under anaerobic conditions, LB medium was turned anoxic by flushing the headspace of sealed tubes with N_2_ gas and by the use of the reducing agent thioglycolate. After 24 hours of growth in the absence of oxygen, we quantified the fluorescence from individual cells of the different constructions by epifluorescence microscopy. Under this condition, fluorescence from most constructions was low, with values being close and not significatively different to the negative control (Fig. 5A). Exception was for the strain carrying carrying pFD145 which showed the highest fluorescence. As expected, the strain carrying a GFP-based plasmid showed a dramatic decrease in fluorescence (compare Fig. 5A with Fig. 3A), as expected from its O_2_ dependency for GFP maturation. It should be noted that strain growths were very low under this condition, probably because of the absence of electron acceptor for respiration and a very low concentration of fermentative products in the LB medium, hindering the production of energy.

In a second set of experiments, an alternative electron acceptor (NO_3_^-^) was added to anoxic LB medium. Under this condition, fluorescence from KOFP-7 increased significantly, being statistically different from the one of the negative control for all strains, except for pFD115 (Fig. 5B and D). The highest per-cell fluorescence intensity was again obtained for the strain carrying pFD145, followed by the strain carrying pFD116, similar to the results obtained in the presence of oxygen. It should be noted that under this condition, the fluorescence from KOFP-7 is 3.2-fold lower than the one measured in the presence of oxygen (compare Fig. 3A and 5B). As expected, GFP-containing cells remained dimmed under this condition (Fig. 5B and D).

In the last set of experiment, every bioreporter was grown without oxygen in LB containing 2% glucose. Fluorescence was generally slightly higher than the ones obtained in anoxic LB alone for most constructions (compare Fig. 5A and C), but lower than the fluorescence obtained from cultures grown in LB-NO_3_^-^ (compare Fig. 5B and C). Under this condition, GFP-containing cells were not fluorescent.

### KOFP-7-based constructions allow cell fluorescence in various bacterial species

**Fig 6:**
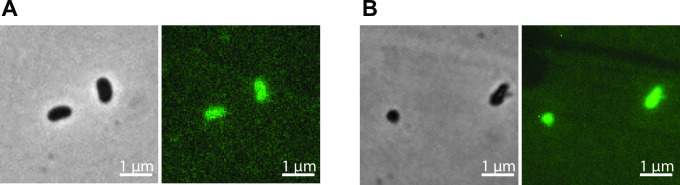
Illustration of the fluorescence of single cells of *V. diazotrophicus* containing pFD145 after growth under oxic (**A**) and anoxic (**B**) conditions

Finally, we sought to demonstrate whether KOFP-7-based constructions can be used in other species than *E. coli*. Therefore, we introduced by conjugation the plasmid pFD145, presenting the most promising results in *E. coli*, in *V. diazotrophicus* NS1, a facultative anaerobe with diazotrophic properties. *V. diazotrophicus* cells carrying pFD145 was monitored both in the presence and absence of oxygen in LB medium and we demonstrated that cells are fluorescent, both in the presence (Fig. 6A) and absence (Fig. 6B) of oxygen.

## DISCUSSION

Fluorescent proteins have been used for decades to unravel a myriad of biological processes and constitute now a standard working tool in molecular biology, cell biology, microbiology, and immunology labs worldwide. The vast majority of these proteins derive from the GFP protein, encoded from the *Aequorea victoria* genome. Many derivatives have been constructed since, differing in their maximum excitation and emission wavelength, their folding speed, their propensity to stay in monomers, or their intrinsic brightness (2). However, they all have in common their O_2_-dependent maturation, required to turn fluorescent (3). This O_2_ dependence represents a major bottleneck in the study of biological processes that take place in the absence of oxygen.

In this study, we present the development of various bacterial bioreporters, which all have in common their O_2_-independent fluorescence. They rely on the use of KOFP-7, a flavin-binding fluorescent protein which has been obtained by genetic engineering and which has been reported to present a higher fluorescence and quantum yield as compared to its parental protein SB2, measured *in vitro* from the purified protein (17). In order to obtain the highest KOFP-7 production and bacterial fluorescence, various genetic circuits controlling *kofp-7* expression were tested, differing in their promoter strength and RBS regions. We demonstrated here that most of the constructions allowed fluorescence from KOFP-7, both under oxic (Fig. 3) and anoxic conditions (Fig. 5). Moreover, these circuits were implemented into various plasmidic backbones, varying in their origin of replications for *E. coli* (colE1 for pFD141 and 148, R6K for pFD115, pFD116, and pFD145, and pBBR1 for pFD149). These plasmids were also chosen, because they are shuttle vectors and can replicate in bacteria outside *E. coli*: *Vibrionaceae* (20, 21) and *Pseudoalteromonaceae* (20) for pFD115, pFD116 and pFD145, *Rhizobiaceae* for pFD141 and pFD148, while pFD149 can maintain in various Gram-negative bacteria.

Among the tested constructions, the pFD145 plasmid showed the most promising results, allowing the highest population and individual cell-based fluorescence (Fig. 3 and 5). The stability of this fluorescence was therefore tested using this construction. We demonstrated here that KOFP-7 is rapidly photobleached, with cells turning almost non-fluorescent after 10 seconds of excitation at 482 +/− 35 nm (Fig. 4). This photobleaching can impede the use of KOFP-7 under specific conditions, but most of the applications are performed with an excitation time below the second (e.g., 100 ms in the present study), decreasing this potential pitfall.

The major advantage of KOFP-7-based bioreporters over GFP-based ones is their capability to fluoresce in the absence of oxygen (14). We demonstrated here that the newly constructed bioreporters are indeed fluorescent in the absence of oxygen (Fig. 5). It should be noted that the measured fluorescence is lower than the one obtained with oxygen (Fig. 3A). Given the O_2_-independent maturation of the fluorochrome, this decrease in fluorescence is likely due to a decrease in the energetic status of the cells when grown in anaerobic conditions as tested in the present study. Indeed, cells were grown in LB, which is an amino acid-rich medium but otherwise rather poor in saccharides (24). In the absence of oxygen or alternative electron acceptor, *E. coli* cells cannot respire and therefore cannot produce ATP by oxidative phosphorylation. In the absence of electron acceptors (Fig. 5A and C), ATP is produced by fermentation. However, amino acids cannot be fermented and the amount of fermentative products is low in LB, limiting this metabolism (24). Therefore, the energetic status of *E. coli* when grown anaerobically in LB is low, probably explaining the low cell fluorescence. In line with this hypothesis, the cell fluorescence increased when cells were grown in anoxic condition in LB supplemented with glucose (as a fermentative product, see Fig. 5C) and especially with NO_3_^-^, which served as electron acceptor for anaerobic oxidative phosphorylation. (Fig. 5B). Under the same conditions, GFP-based bioreporters remained dimmed, demonstrating that the increase of KOFP-7-based fluorescence is not due to the addition of oxygen during the injection of NO_3_^-^. The lower fluorescence obtained when NO_3_^-^ is used as an alternative electron acceptor, as compared with aerobic growth, is probably due to the lower amount of ATP produced by anaerobic oxidative phosphorylation and the fact that O_2_ remains the best electron acceptor to produce ATP.

Finally, we anticipate that KOFP-7-based constructions can be used to study metabolic and physiological processes in the absence of O_2_. As a proof of concept, we monitored the expression of pFD145 in *V. diazotrophicus*, a facultative anaeorbe. We could demonstrate that KOFP-7 is produced under anoxic condition and allows cell fluorescence in bacteria other than *E. coli* (Fig. 6). KOFP-7-based constructions are therefore useful tools, functional in various bacterial species and irrespective of the oxygen availability.

To conclude, we present here the development of new set of bioreporters, allowing illumination of bacteria by fluorescence in the absence of oxygen. The developed plasmids can be used in a broad range of Gram-negative bacteria and can be used for the study of physiological processes occurring in the absence of oxygen. The current limitation of KOFP-7 is its rapid photobleaching, compensated by its fast maturation time, and its lower intrinsic fluorescence. However, given the recent advances in genetic engineering, derivatives of KOFP-7 or other FbFPs with increased fluorescence will likely emerge in the nearer future, in order to shed light onto the still under-explored world of anoxic environments.

## MATERIALS AND METHODS

### Strain and culture conditions

*Escherichia coli* strains and *V. diazotrophicus* NBRC 103148 strains were routinely grown in LB at 37°C and 30°C, respectively. Some experiments using *V. diazotrophicus* were performed in a Modified Diazotrophic Vibrio (MDV) medium defined elsewhere (25). In order to create anoxic media, 20 mL LB was prepared in 18 cm glass tubes (Dutscher, ref 508232), supplemented with sodium thioglycolate (T0632, Sigma-Aldrich, 10 mg/L final concentration), closed with a sterile blue rubber stopper, and sealed with an aluminum capsule. The headspace was flushed for 2 minutes with either argon or nitrogen gas immediately after autoclaving, when the temperature was still at 80°C. If necessary, agar (15 g/L) or glucose (20 g/L) was added before autoclaving. Absence of oxygen was controlled by adding resazurin (500 µg/L final concentration from a 500 mg/L stock solution) in the media before autoclaving. If necessary, trimethoprim (Trim, 10 µg/mL), kanamycin (50 µg/mL), diaminopimelic acid (DAP, 0.3 mM), NaNO_3_ (4.67 g/L from an autoclaved 466.7 g/L stock solution), 20 g/L were added after autoclaving.

For anoxic growth, one colony of each strain was grown overnight in 100 mL Erlenmeyer containing 20 mL LB + antibiotic. The next morning, anoxic LB was inoculated with 100 µL of overnight cultures and the antibiotic using sterile syringes and needles. If necessary, 200 µL of NaNO_3_ was added at the same time. Tubes were incubated for 24 hours before fluorescence quantification as described below. The strains, plasmids, and primers used in this study can be found in Tables S1 to S3, respectively.

### Construction of plasmids with constitutive *kofp-7* expression

A synthetic sequence containing the Open Reading Frame (ORF) of KOFP-7 was obtained by GeneArt (ThermoFisher Scientific). This sequence contained several attributes, allowing the construction of various plasmids differing in their RBS and promoter sequences, thanks to restriction sites flanking the to-be-cloned region or by Gibson Assembly (using the NEBuilder Hifi DNA Assembly Master Mix, NEB). pFD115 was obtained by replacing the RBS region and *gfp* gene of pFD086 (26) with a fragment containing the RBS of pOT1e (27) and the *kofp-7* gene, using SphI + BamHI. pFD116 plasmid was obtained by replacing the *gfp* gene with the *kofp-7* gene, using SphI + BamHI. pFD141 was obtained by Gibson Assembly, replacing the *mcherry* gene of pMG103-nptII-mcherry (28) with the *kofp-7* gene, using primers 220415–220418. pFD145 was obtained by Gibson Assembly, replacing the P*_lac_* promoter of pFD116 with the P*_nptII_* promoter of pFD141, using 230214–230217. pFD148 was obtained by replacing the P*_nptII_* promoter of pFD141 with the P*_lac_* promoter of pFD116 using XbaI + SphI. pFD149 was obtained inserting the P*_nptII_* promoter of pFD145 (using XhoI + XbaI) in pFD147 (using SalI + XbaI). pFD150 was obtained by digesting pFD116 and pFD085 with XbaI + PstI, replacing the promoterless *gfp* gene by *kofp-7i*. All constructions were verified by PCR and, when Gibson Assembly was done, the absence of mutations, which might have occurred during PCR amplification, has been verified by sequencing. All *in silico* plasmid maps are available upon request.

### Strain constructions

*E. coli* strains were transformed with the different constructed plasmids using heat-shock transformation. *V. diazotrophicus* NS1 was modified by conjugation, using a recently published protocol (25). Briefly, 200 µL of an overnight culture of *E. coli* β-3914 donor strain (29) carrying the desired plasmid was centrifuged. Eight hundred microliters of an overnight culture of *V. diazotrophicus* was added to the *E. coli* pellet, without resuspending the pellet. After a second centrifugation, cells were resuspended in 20 µL LB and spotted on a 0.2 µm acetate filter placed on an LB + DAP plate. After 24 hours of growth at 30°C, the filter was removed, washed with 1 mL LB, and plated on LB plates supplemented with the desired antibiotic. Plates were incubated overnight at 30°C.

### Fluorescence quantification by fluorimetry

One colony of each strain containing plasmids allowing constitutive KOFP-7 production was grown overnight in LB + antibiotic. The next morning, OD_600nm_ was measured, 1 mL of each culture was centrifuged, the pellet was resuspended with 1 mL of minimum fluorescent medium [1 L distilled water supplemented with 10 g glucose, 0.1519 g NH_4_Cl, 0.028 g K_2_HPO_4_, 5 g NaCl, 0.5 g yeast extract, and 0.1 g tryptone [modified from reference (30)]. The pH was adjusted to 7 with a solution of HCl (0.2 M) or NaOH (0.2 M), which displays a lower autofluorescence than LB (30), and further diluted with the same medium to reach OD 0.1. Two hundred microliters of these OD-adjusted cultures were inoculated in 96-well black polystyrene plates (ref 655900, Greiner Bio-One) in quadruplicate and fluorescence was measured using the TECAN Infinite M1000, set with an excitation wavelength of 450 nm and an emission wavelength of 496 nm. Wells filled with minimum fluorescent medium + antibiotic without bacteria were used as a control to remove the fluorescence background of the medium. Microplates were incubated at 37°C with shaking, and fluorescence was measured every hour over 5 hours.

Every experiment was done in biological triplicate (3 independent days), with four technical replicates per biological replicate, every biological replicate giving a similar result. Results from these 12 replicates were pooled to produce Fig. 2.

### Fluorescence quantification by microscopy

Cultures were grown overnight under aerobic conditions or for 24 hours in anoxia. Aliquots of these cultures were subsequently used for fluorescence microscopy. Briefly, agarose patches were prepared by melting 1% agarose in water, followed by incubation at 50°C to equilibrate the temperature. Six hundred microliters of molten agarose was deposited onto a microscopy slide, and a second microscopy slide was immediately added on top of the liquid. Ten minutes later, the patch was recovered by sliding the two microscopy slides and drops of the overnight culture were deposited on the agarose patch before being covered with a coverslip. Pictures were acquired from the overnight cultures using an Eclipse Ni-E microscope (Nikon) equipped with a 100× Apochromat oil objective, an FITC-3540C filter cube (482 +/− 35 nm excitation) at 100 ms exposure time. Fluorescence of single cells was quantified using an in-house written Matlab script (23) from at least eight independent pictures. Every condition was tested at least in duplicates, each replicate consisting in at least eight independent images. Presented here are results from pooled replicates.

In order to observe KOFP-7-based fluorescence in *V. diazotrophicus*, we grew cells *V. diazotrophicus* in LB + Trim. One hundred microliters of these overnight precultures were used to inoculate 20 mL LB + Trim either under aerobic condition (in 100 mL Erlenmeyer with shaking) or under anaerobic condition (18 cm glass tubes as described above) and 200 µL of NaNO_3_^-^. After 12 hours (for aerobic cultures) or 24 hours (for anoxic cultures), drops of the cultures were deposited onto agarose patch and immediately monitored by epifluorescence microscopy.

### Statistical analyses

All statistical analyses were performed using GraphPad Prism. An ordinary one-way analysis of variance with Tukey test was performed for all comparisons. *P*-values <0.05 were considered statistically significant.

## SUPPLEMENTAL MATERIAL

Tables S1 to S3 spectrum.04091-23-s0001.pdf Strains, plasmids, and primers used in this study.

ASM does not own the copyrights to Supplemental Material that may be linked to, or accessed through, an article. The authors have granted ASM a non-exclusive, world-wide license to publish the Supplemental Material files. Please contact the corresponding author directly for reuse.
