# Supplementary material for: Development and utilization of new O2-independent bioreporters
Source: Microbiol Spectr. 2024 Mar 5;12(4):e04091-23. doi: 10.1128/spectrum.04091-23 (PMC10986488; doi:10.1128/spectrum.04091-23)
Supplement: Tables S1 to S3 — Strains, plasmids, and primers used in this study. [file spectrum.04091-23-s0001.pdf]

Table S1. Strains used in this study.

Strain collection number (US2B lab collection)	Strain name	Characteristics	References
Escherichia coli			
204	DH5α λpir	*sup E44, ΔlacU169 (ΦlacZΔM15), recA1, endA1, hsdR17, thi-1, gyrA96, relA1, λpir phage lysogen*	Lab collection
206	GEB883 + pEVS104	Strain GEB883 (*1*) containing pEVS104 (*2*)	(*3*)
207	β-3914	F^{-}, RP4-2-Tc::Mu, Δ*dapA*::(*erm-pir*) *gyrA462 zei-298::*Tn*10*, Km^{R}, Em^{R}, Tc^{R}, DAP^{-}	(*4*)
254	DH5α λpir pFD086	Derivative of strain 204 containing pFD086	(*3*)
349	DH5α λpir pFD115	Derivative of strain 204 containing pFD115	This study
351	DH5α λpir pFD116	Derivative of strain 204 containing pFD116	This study
444	DH5α λpir pFD141	Derivative of strain 204 containing pFD141	This study
489	DH5α λpir pFD145	Derivative of strain 204 containing pFD145	This study
490	DH5α λpir pFD146	Derivative of strain 204 containing pFD146	This study
491	DH5α λpir pFD147	Derivative of strain 204 containing pFD147	This study
505	DH5α λpir pFD148	Derivative of strain 204 containing pFD148	This study
506	DH5α λpir pFD149	Derivative of strain 204 containing pFD149	This study
529	DH5α λpir pFD149	Derivative of strain 204 containing pFD150	This study
V. diazotrophicus			
295	*V. diazotrophicus* NBRC 103148	Strain isolated from urchin gut	(*5*)
522	*V. diazotrophicus* NBRC 103148 pFD145	Derivative of strain 295 carrying pFD145	This study

1. A. N. Nguyen *et al.*, *csrB* Gene Duplication Drives the Evolution of Redundant Regulatory Pathways Controlling Expression of the Major Toxic Secreted Metalloproteases in *Vibrio tasmaniensis* LGP32. *mSphere* **3**, (2018).
2. E. V. Stabb, E. G. Ruby, RP4-based plasmids for conjugation between *Escherichia coli* and members of the *Vibrionaceae*. *Method Enzymol* **358**, 413-426 (2002).

3. A. Morot *et al.*, Virulence of *Vibrio harveyi* ORM4 towards the European abalone *Haliotis tuberculata* involves both quorum sensing and a type III secretion system. *Environ Microbiol* **23**, 5273-5288 (2021).
4. F. Le Roux, J. Binesse, D. Saulnier, D. Mazel, Construction of a *Vibrio splendidus* mutant lacking the metalloprotease gene vsm by use of a novel counterselectable suicide vector. *Appl Environ Microbiol* **73**, 777-784 (2007).
5. M. L. Guerinot, D. G. Patriquin, N_2-fixing vibrios isolated from the gastrointestinal tract of sea urchins. *Can J Microbiol* **27**, 311-317 (1981).

Table S2. Plasmids used in this study. All *in silico* plasmid sequences and maps are available upon request

Plasmid name	Plasmid characteristics	Reference
pGEM-T	Cloning vector. *lacZ*. Amp^R	Promega
pEVS104	Conjugative helper plasmid. $oriV_{R6K}$ $oriT_{RP4}$. Km^R	(*1*)
pFD085	Replicative plasmid for *Vibrio*, containing a promoterless *gfp* gene. $Trim^R$	(*2*)
pFD086	Replicative plasmid for *Vibrio*, containing the P_{lac} promoter upstream of the *gfp* gene. $Trim^R$	(*2*)
pOT1e	Broad-host-range plasmid, containing a promoterless *gfp* gene. ori pBBR1. Gm^R	(*3*)
pMG103-nptII-mcherry	Shuttle vector replicating in *E. coli and Rhizobiaceae*. Contains the P_{nptII} promoter controlling *mcherry* expression. Km^R	(*4*)
pFD115	Derivative of pFD086 containing the RBS region of pOT1e and in which the *gfp* gene has been replaced by *kofp-7*. $Trim^R$	This study
pFD116	Derivative of pFD086 in which the *gfp* gene has been replaced by *kofp-7*. $Trim^R$	This study
pFD141	Derivative of pMG103-nptII-mcherry in which the RBS-*mcherry* fragment has been replaced by RBS-*kofp-7* from pFD116. Km^R	This study
pFD145	Derivative of pFD116 in which the P_{lac} promoter has been replaced by P_{nptII} from pFD141. $Trim^R$	This study
pFD148	Derivative of pFD141 in which the P_{nptII} promoter has been replaced by P_{lac} from pFD116. Km^R	This study
pFD149	Derivative of pFD147 in which the P_{nptII} promoter has been inserted. Km^R	This study
pFD150	Derivative of pFD085 in which the *gfp* gene has been replaced by the *kofp-7* gene. $Trim^R$	This study

1. E. V. Stabb, E. G. Ruby, RP4-based plasmids for conjugation between *Escherichia coli* and members of the *Vibrionaceae*. *Method Enzymol* **358**, 413-426 (2002).
2. A. Morot *et al.*, Virulence of *Vibrio harveyi* ORM4 towards the European abalone *Haliotis tuberculata* involves both quorum sensing and a type III secretion system. *Environ Microbiol* **23**, 5273-5288 (2021).
3. D. Allaway *et al.*, Use of differential fluorescence induction and optical trapping to isolate environmentally induced genes. *Environ Microbiol* **3**, 397-406 (2001).
4. K. Bonaldi *et al.*, The Nod Factor-Independent Symbiotic Signaling Pathway: Development of *Agrobacterium rhizogenes*-Mediated Transformation for the Legume *Aeschynomene indica*. *Mol Plant Microbe In* **23**, 1537-1544 (2010).

Table S3. Primers used in this study

Primer number	Primer name	Primer sequence	Target
191103	EVS58	CATGATCGAGCTTAATTCTGGAAGGCAGTACACCTTGATAG	Primer annealing within pFD086 and derivatives. Used in combination with 210214 to verify the correct constructions of pFD115 and pFD116
191116	T7	TAATACGACTCACTATAGGG	Primer annealing on many plasmids used in this study.
210214	KOFP7-R	CCAGCTCACGCATTCTTTC	Primer annealing close to the end of the *kofp-7* gene, facing towards ATG. Used to verify many constructions of this study
220415	KOFP7_for_pMG103-F	GATCAAGATCTCTAGAGTCGACAATAATTTTGTTTAACTTTAAGAAGGAG	Primer annealing upstream of the ATG of *kofp-7* in pFD116, facing inwards. Used in combination with 220416, 220417 and 220418 to construct pFD141
220416	KOFP7_for_pMG103-R	AATTCGATATCggatccTTACTGGCGTCGC	Primer annealing at the end of the stop codon of *kofp-7* in pFD116, facing inwards. Used in combination with 220415, 220417 and 220418 to construct pFD141
220417	Vec-pMG103-KOFP7-F	GTAAggatccGATATCGAATTCGTAATCATGGTCATAGCTG	Primer annealing at the end of the stop codon of *mcherry* in pMG103-npt2-mcherry, facing outwards. Used in combination with 220415, 220416 and 220418 to construct pFD141
220418	Vec-pMG103-KOFP7-R	TCGACTCTAGAGATCTTGATCCCCTGCGCC	Primer annealing upstream of the ATG of *mcherry* in pMG103-npt2-mcherry, facing outwards. Used in combination with 220415, 220416 and 220417 to construct pFD141
230214	pFD116_vec-F	GATATACATATGAGTCCGATGATTAATGCCAAACT	Primer annealing at the RBS region of *kofp-7* within pFD116 (strain 351), facing towards ATG. Used in combination with 230215, 230216 and 230217 to construct pFD145
230215	pFD116_vec-R	TGGCATGCCTCGAGGAAAAAGACCCTTCATAAATGAAGG	Primer annealing at the beginning of P_{lac} in pFD116 (strain 351), facing outwards. Used in combination with 230214, 230216 and 230217 to construct pFD145
230216	pnpt2_frag_F	CCTCGAGGCATGCCACGCTGC	Primer annealing at the beginning of the P_{nptII} promoter of pFD141 (strain 444), facing inwards.

			Used in combination with 230214, 230215 and 230217 to constrct pFD145
230217	pnpt2_frag_R	TAATCATCGGACTCATATGTATATCTCCTTCTTAAAGT	Primer annealing at the end of the P_{nptII} promoter of pFD141 (strain 444), facing inwards. Used in combination with 230214, 230215 and 230216 to construct pFD145
230301	Plac-SphI-F	ATGCGCATGCGGTACCcTCGAGCAAGACGTTTCCC	Primer annealing at the beginning of the P_{lac} promoter of pFD086 and derivatives, facing inwards. Used in combination with 230302 to construct pFD148
230302	Plac-XbaI-R	ATGCTCTAGAGCAAGCTTGAGTATTCTATAG	Primer annealing at the end of the P_{lac} promoter of pFD086 and derivatives, facing inwards. Used in combination with 230301 to construct pFD148
